# Identification and validation of biomarkers related to lipid metabolism in osteoarthritis based on machine learning algorithms

**DOI:** 10.1186/s12944-024-02073-5

**Published:** 2024-04-18

**Authors:** Hang Li, Yubao Cui, Jian Wang, Wei Zhang, Yuhao Chen, Jijun Zhao

**Affiliations:** 1 Wuxi Medical Center, Nanjing Medical University, No. 299 Qing Yang Road, Wuxi, Jiangsu 214023 China; 2 Clinical Research Center, The Affiliated Wuxi People’s Hospital of Nanjing Medical University, No. 299 Qing Yang Road, Wuxi, 214023 Jiangsu China; 3 Department of Orthopedic, The Affiliated Wuxi People’s Hospital of Nanjing Medical University, No. 299 Qing Yang Road, Wuxi, Jiangsu 214023 China

**Keywords:** Osteoarthritis, Lipid metabolism, Bioinformatics analysis, Hub genes, Machine learning, Immune infiltration

## Abstract

**Background:**

Osteoarthritis and lipid metabolism are strongly associated, although the precise targets and regulatory mechanisms are unknown.

**Methods:**

Osteoarthritis gene expression profiles were acquired from the GEO database, while lipid metabolism-related genes (LMRGs) were sourced from the MigSB database. An intersection was conducted between these datasets to extract gene expression for subsequent differential analysis. Following this, functional analyses were performed on the differentially expressed genes (DEGs). Subsequently, machine learning was applied to identify hub genes associated with lipid metabolism in osteoarthritis. Immune-infiltration analysis was performed using CIBERSORT, and external datasets were employed to validate the expression of these hub genes.

**Results:**

Nine DEGs associated with lipid metabolism in osteoarthritis were identified. UGCG and ESYT1, which are hub genes involved in lipid metabolism in osteoarthritis, were identified through the utilization of three machine learning algorithms. Analysis of the validation dataset revealed downregulation of UGCG in the experimental group compared to the normal group and upregulation of ESYT1 in the experimental group compared to the normal group.

**Conclusions:**

UGCG and ESYT1 were considered as hub LMRGs in the development of osteoarthritis, which were regarded as candidate diagnostic markers. The effects are worth expected in the early diagnosis and treatment of osteoarthritis.

**Supplementary Information:**

The online version contains supplementary material available at 10.1186/s12944-024-02073-5.

## Introduction

Osteoarthritis (OA) is a common degenerative disease that mainly causes clinical symptoms such as joint pain, swelling, and deformity [1]. Risk factors associated with OA encompass age, gender, obesity, and genetics [2]. With the aging of the population and the increase in the average life expectancy of human beings, the incidence of osteoarthritis remains high, which imposes a huge economic burden on individuals as well as society. Although arthroplasty is an effective treatment for advanced OA, it is marred by issues such as limited prosthesis lifespan and restricted postoperative joint function. Hence, early prevention and intervention assume paramount significance [3].

The incidence of knee OA is notably higher in obese patients than in normal subjects, and obesity also increases the risk of developing hand joint OA [4]. These findings collectively imply that, in addition to mechanical factors, metabolic factors play a role in the development of OA [5]. Lipid metabolism, as a common pathway in metabolic diseases and OA, may have direct systemic effects on joints [6]. Osteoarthritic cartilage contains large amounts of lipid deposits, especially in chondrocytes. It has been shown that the total fatty acid and arachidonic acid content of articular cartilage samples gradually increases with the progression of OA [7]. Irregular lipid accumulation in OA chondrocytes may be the cause of the onset and progression of OA [8]. Furthermore, molecular markers of lipid peroxidation are present in OA chondrocytes, suggesting that the pathogenesis of OA may also be related to lipid oxidation [9]. Many changes in lipid metabolism, including cartilage, subchondral bone, and periosteum, are involved in the pathogenesis of OA. These changes interact with inflammatory mediums and affect the development of lesions. Adjusting lipid metabolism pathways through diet or drugs can prevent cartilage degradation and delay the progression of OA [10]. However, the precise mechanisms through which lipid metabolism influences osteoarthritis remain unclear and need to be further explored.

Currently, microarray technology and integrated bioinformatics analysis have been widely used to identify potential biomarkers and their role in various diseases [11]. GEO is an international public repository that collects and organizes various high-throughput sequencing data. The MigSB database includes numerous annotated gene sets that can be searched and downloaded using relevant keywords. These gene sets are crucial in the field of biomedicine and provide valuable data for bioinformatics research. In this study, we screened hub genes of lipid metabolism in OA by bioinformatics analysis technology combined with machine learning algorithms. These genes were subsequently validated using independent validation datasets, to elucidate the regulatory mechanisms underpinning lipid metabolism in OA development and generating innovative insights for the clinical diagnosis and treatment of OA.

## Materials and methods

### Data collection

 Osteoarthritis gene expression probe matrix files and platform files were extracted from the GEO database (http://www.ncbi.nlm.nih.gov/geo/), with the qualifying criteria of (i) Osteoarthritis; (ii) Expression profiling by array; and (iii) Homo. The final datasets GSE48556 and GSE206848 were selected. GSE48556 consists of 33 healthy controls and 106 osteoarthritis patients on GPL6947 (Illumina HumanHT-12 V3.0 expression beadchip) [12]. GSE206848 consists of 7 healthy controls and 7 osteoarthritis patients data on platform GPL570 (Affymetrix Human Genome U133 Plus 2.0 Array). In addition, 776 lipid metabolism-related genes (LMRGs) were sorted from the lipid metabolism pathways, which were downloaded from the Molecular Signatures Database (MSigDB, https://www.gsea-msigdb.org/gsea/msigdb) (Additional Table 1).

### Data processing

The expression matrix and clinical information of the dataset GSE48556 were extracted using R software (https://www.bioconductor.org/). The gene expression matrix was standardized and analyzed by “normalizeBetweenArrays” in the “limma” package, and the standardized gene expression matrix was output.

### Screening of osteoarthritis lipid metabolism differential genes

The gene expression matrix after normalization was subjected to differential analysis by using the “limma” package in R software, which was analyzed by using “ggplot2” in R software. The screening criteria were fold change (FC) > 1.2 and adj.P.value < 0.05 for statistically significant. The “ggplot2” package in R software was used to create a volcano map of DEGs.

### Functional enrichment analysis

Gene Ontology (GO) enrichment analysis and Kyoto Encyclopedia of Genes and Genomes (KEGG) enrichment analysis are commonly used in bioinformatics, which can explore the relationship between genes and enrichment-related functions. GO consists of three parts: biological process, cellular component, and molecular function. The GO and KEGG enrichment analysis of DEGs was performed by using the “cluster Profiler” package in R software with the qualifications of p-value filter = 0.05 and q-value filter = 1, and the results of the enrichment analysis were presented in the form of strip plots.

### Immune infiltration analysis

CIBERSORT is the most cited immune cell infiltration estimation analysis tool, which is based on the principle of linear support vector regression to deconvolve the expression matrix of human immune cell subtypes. We utilized CIBERSORT to estimate the proportion of immune cell types between the healthy control group and the osteoarthritis group.

### Osteoarthritis lipid metabolism differential gene screening

The intersection of osteoarthritis DEGs and lipid metabolism-related genes was obtained by using R software to obtain the osteoarthritis lipid metabolism-related genes, and the Wayne diagram was produced by using the “venn” package in R software.

### Machine learning algorithms for screening hub genes

Three machine learning algorithms, namely, least absolute shrinkage and selection operator (LASSO) regression, support vector machine-receursive feature elimination (SVM-RFE) algorithm, and random forest (RF) algorithm, were applied to screen the hub genes. LASSO regression searches for the smallest classification error λ to determine variables. It is mainly used for screening feature variables and constructing the best classification model. SVM-RFE is a machine learning method based on the support vector machine, which searches for the optimal variable by removing the feature vectors generated by SVM. Predictive performance is estimated by 10-fold cross-validation. Random forest is a machine learning algorithm constructed by the decision tree algorithm, widely used to solve regression and classification problems. It performs well under low sample size situations [13]. The results of the three machine learning algorithms were intersected to screen out the hub genes of lipid metabolism in osteoarthritis. Subsequently, these results were visualized and utilized for further analysis.

### Validation of hub genes in the validation dataset

The expression matrix of the validation dataset GSE206848 was extracted using R software, and two osteoarthritis samples with obvious abnormal results were excluded. The expression profiles of the hub genes associated with lipid metabolism in osteoarthritis were then visualized within the validation set. Hub genes demonstrating statistically significant differences in expression were identified as the definitive hub genes.

## Results

### Osteoarthritis lipid metabolism differential genes

**Fig. 1:**
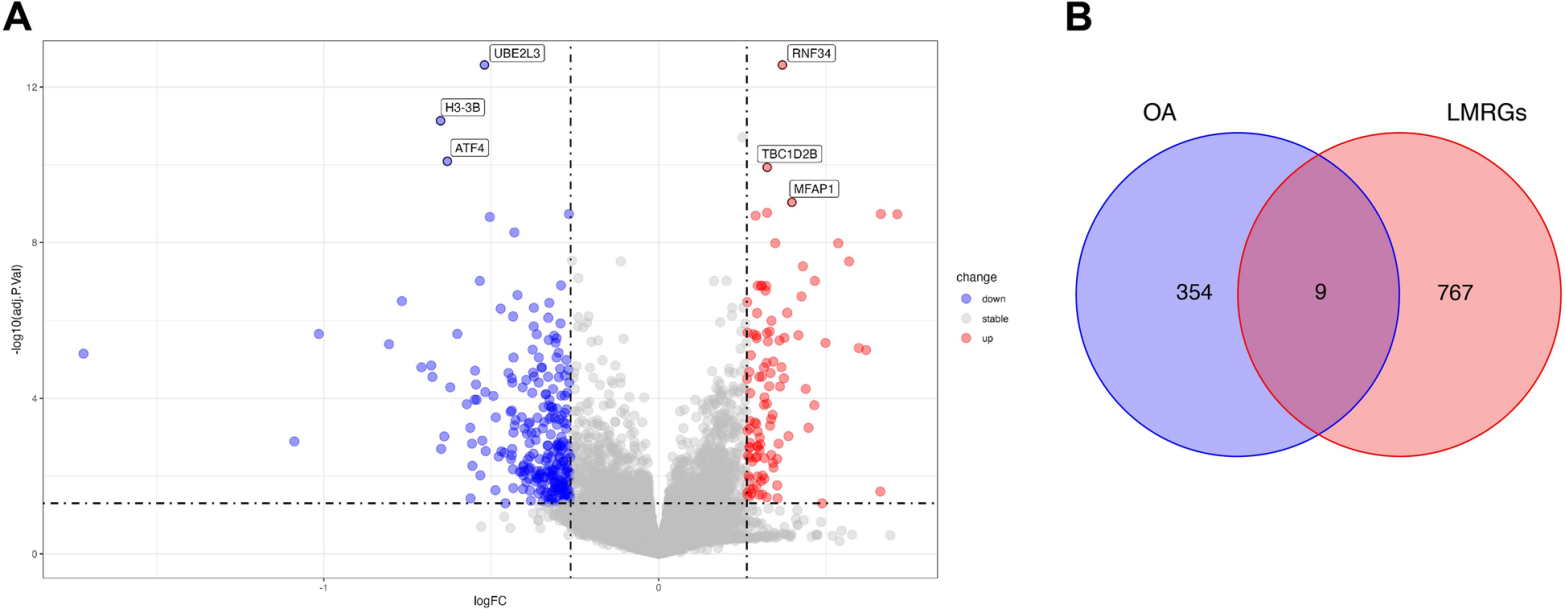
Differentially expressed genes of osteoarthritis and lipid metabolism-related genes (**A**) Volcano plot for the DEGs identified from GSE48556. Red and blue plot triangles represent DEGs with upregulated and downregulated gene expression, respectively. **B **Venn diagram of osteoarthritis DEGs and lipid metabolism gene: red represents lipid metabolism-related genes, blue represents osteoarthritis DEGs. DEGs, differentially expressed genes

The batch effect was removed from the dataset GSE48556, and adj. *P*.value < 0.05 and |log2FC| > log_2_1.2 were used as the screening criteria for the analysis of variance. Subsequently,363 DEGs were finally obtained (Fig. 1A). The intersection of osteoarthritis DEGs and 776 lipid metabolism-related genes obtained was taken to obtain 9 hub genes (Fig. 1B).

### GO and KEGG enrichment analysis

**Fig. 2:**
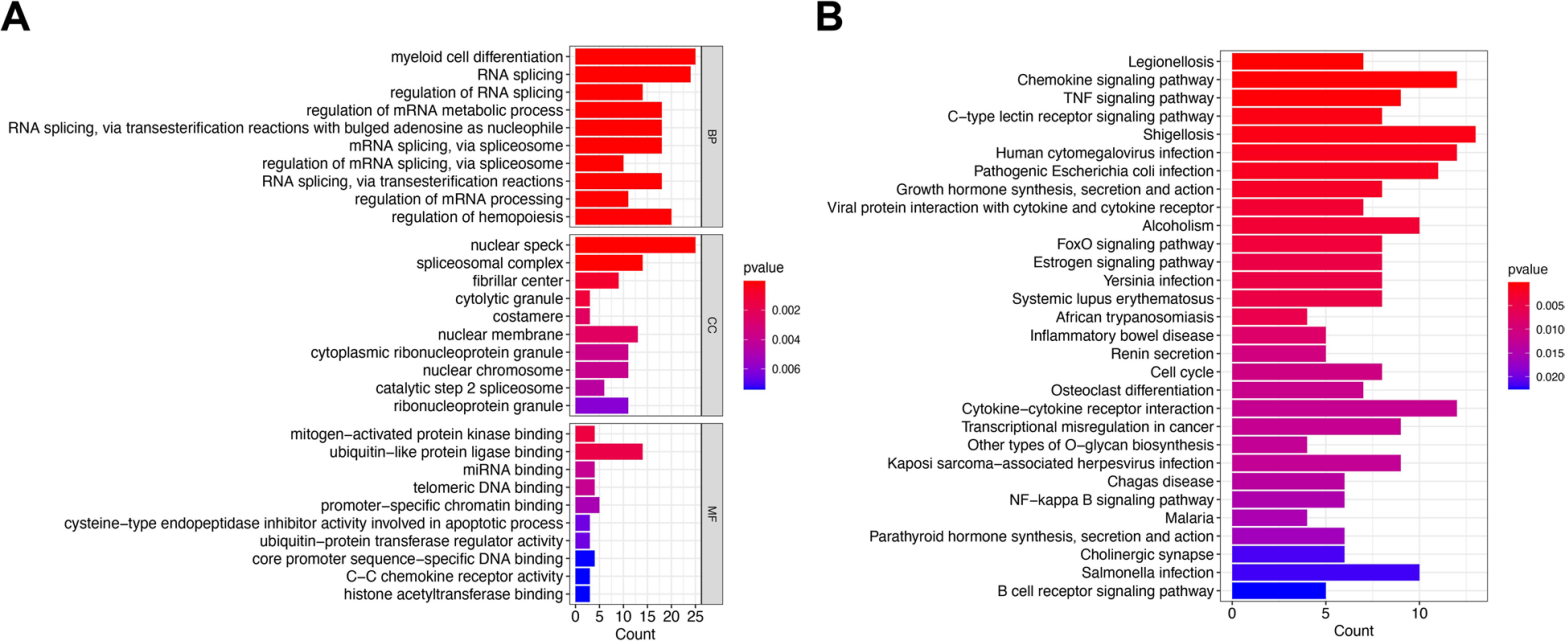
Functional enrichment analysis results. **A **GO analysis of the 363 osteoarthritis DEGs. **B **KEGG pathway analysis of the 363 osteoarthritis DEGs

The 363 osteoarthritis DEGs were subjected to GO and KEGG enrichment analysis. GO enrichment analysis showed that these genes were mainly enriched in myeloid cell differentiation, RNA splicing, regulation of hemopoiesis, and other biological processes. In cellular components, they were mainly present in nuclear specks, spliceosomal complexes and nuclear membranes. In molecular functions, they were mainly enriched in ubiquitin-like protein ligase binding (Fig. 2A).KEGG enrichment analysis showed that these genes were mainly activated in the chemokine signaling pathway, TNF signaling pathway, and C-type lectin receptor signaling pathway (Fig. 2B).

### Immune infiltration analysis

**Fig. 3:**
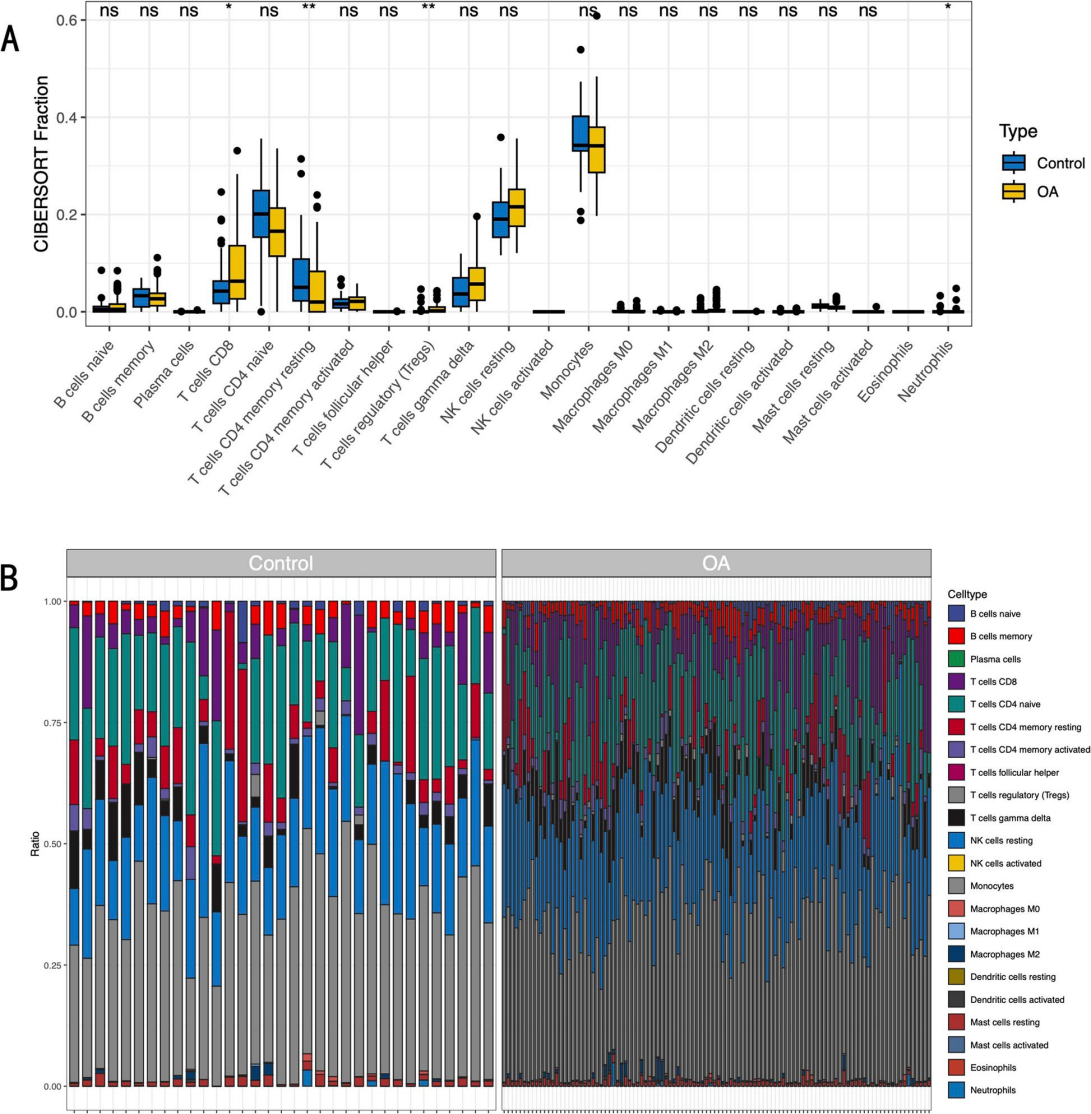
Results of immune infiltration analysis. **A **Comparison regarding the proportion of 22 kinds of immune cells between OA and control groups visualized by the boxplot. *p < 0.05; **p < 0.01; ***p < 0.001. A value of *p* < 0.05 was considered to be statistically significant. **B **The proportion of 22 kinds of immune cells in different samples was visualized from the burplot

CIBERSORT immune infiltration analysis was performed on GSE48556. The results showed that the number of CD8 + T cells and regulatory T cells in osteoarthritis patients was higher than that in the control group, and the number of CD4 + memory T cells was lower than that in the control group (Fig. 3).

### GO and KEGG enrichment analysis of 9 hub genes

**Fig. 4:**
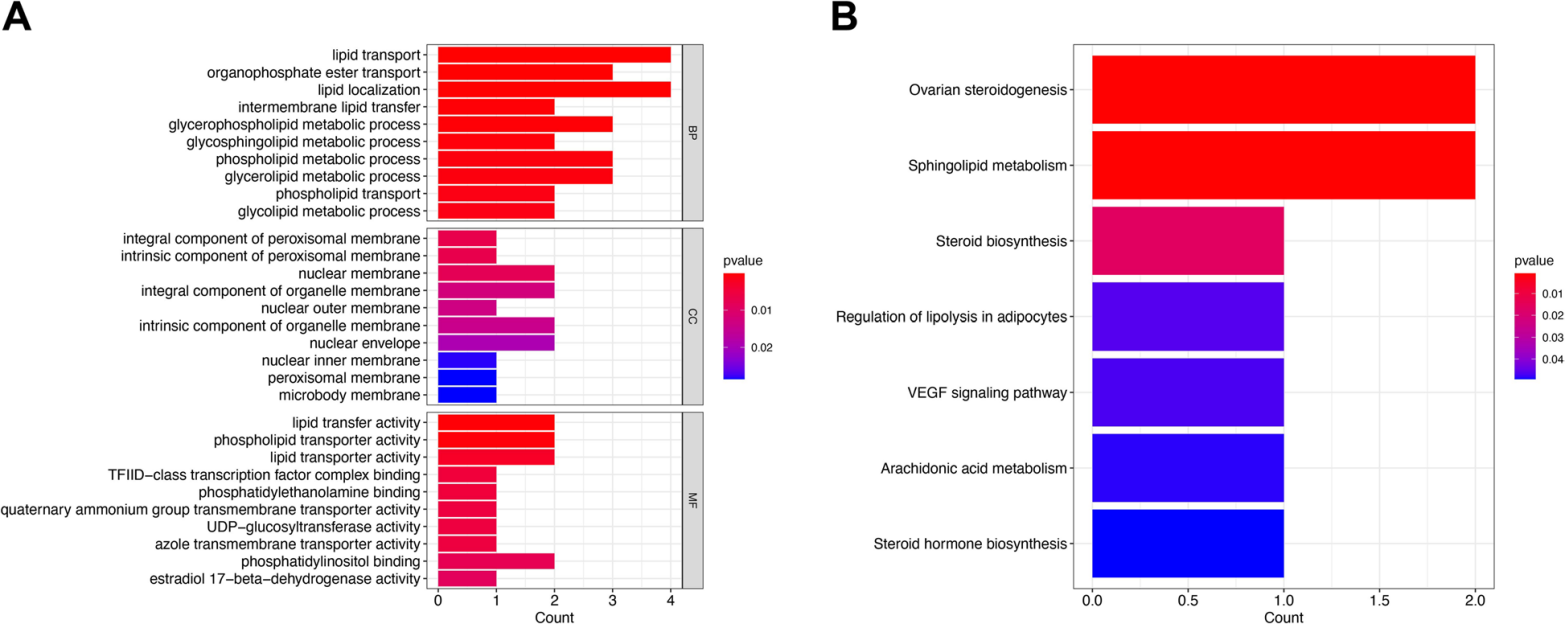
GO and KEGG enrichment analysis results. **A **GO pathway analysis of the 9 hub genes. **B **KEGG pathway analysis of the 9 hub genes

GO and KEGG enrichment analysis was performed on 9 hub genes. GO enrichment analysis showed that these several hub genes were mainly enriched in biological processes such as lipid transport, organophosphate ester transport, and lipid localization; in cellular components, they were mainly related to the peroxisomal membrane, nuclear membrane, and organelle membrane. In molecular functions, they are mainly related to lipid transfer activity, phospholipid transporter activity, and lipid transporter activity (Fig. 4A). KEGG enrichment analysis showed that these genes are mainly activated in the pathways of ovarian steroidogenesis, sphingolipid metabolism, steroid biosynthesis, and other pathways (Fig. 4B).

### Machine learning algorithms

**Fig. 5:**
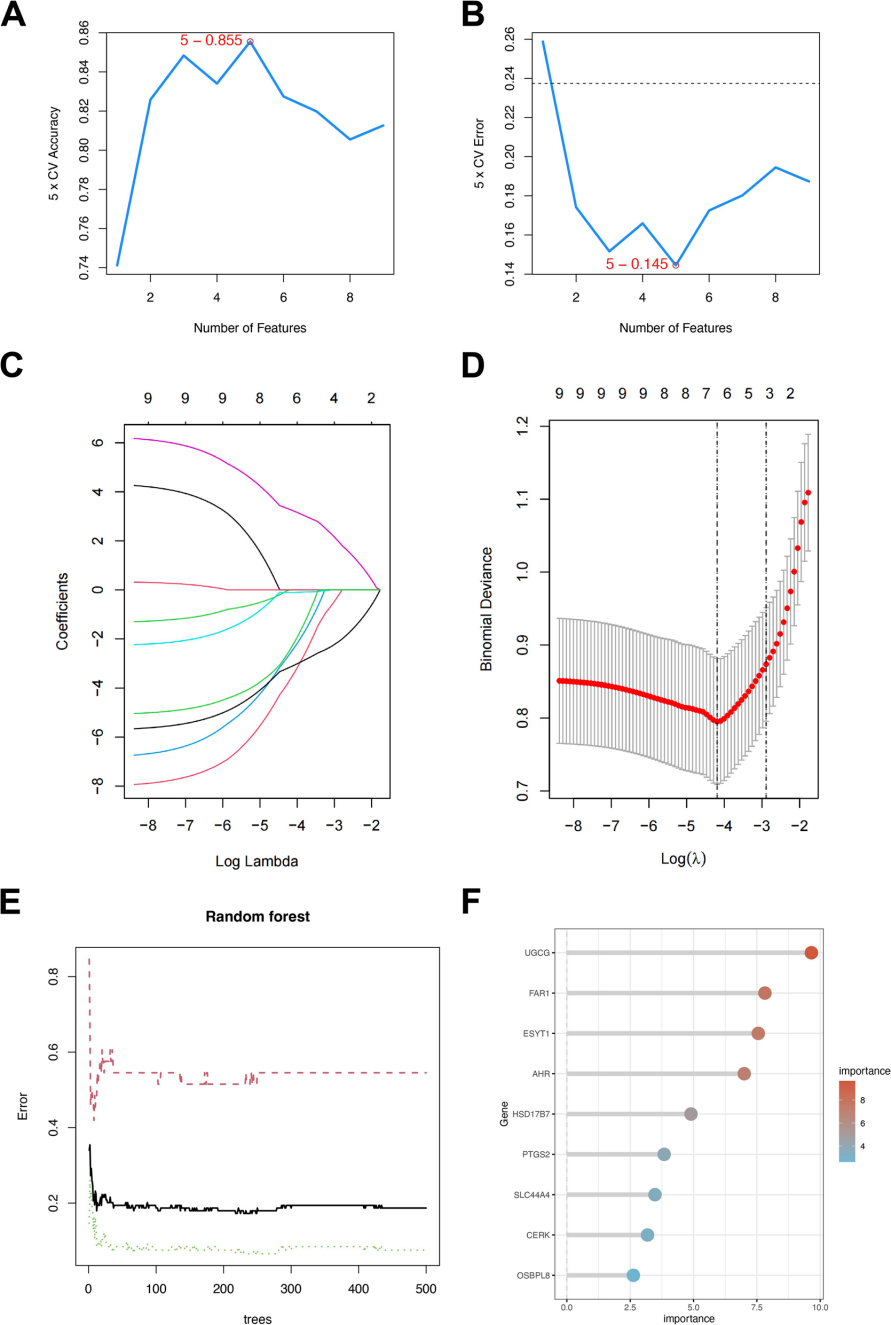
Screening of hub genes by three machine learning algorithms. **A**,** B **SVM-RFE algorithm for feature gene selection. **C**,** D** LASSO algorithm. Vertical dashed lines are plotted at the best lambda. **E**, **F **The random forest algorithm shows the error in OA. The control group and genes are ranked based on the importance score

The 9 hub genes were identified as 5, 6, and 9 hub genes using three machine learning algorithms, SVM-RFE, LASSO, and RF (Fig. 5). 4 hub genes (UGCG, ESYT1, PTGS2, and CERK) were finally identified.

### Osteoarthritis lipid metabolism hub genes

The nine osteoarthritis lipid metabolism differential genes were identified as 5, 6, and 9 hub genes using three machine learning algorithms, SVM-RFE, LASSO, and RF, respectively. Ultimately, 4 hub genes (UGCG, ESYT1, PTGS2, and CERK) were identified as vital players in osteoarthritis lipid metabolism.

### Validation results of the validation set

**Fig. 6:**
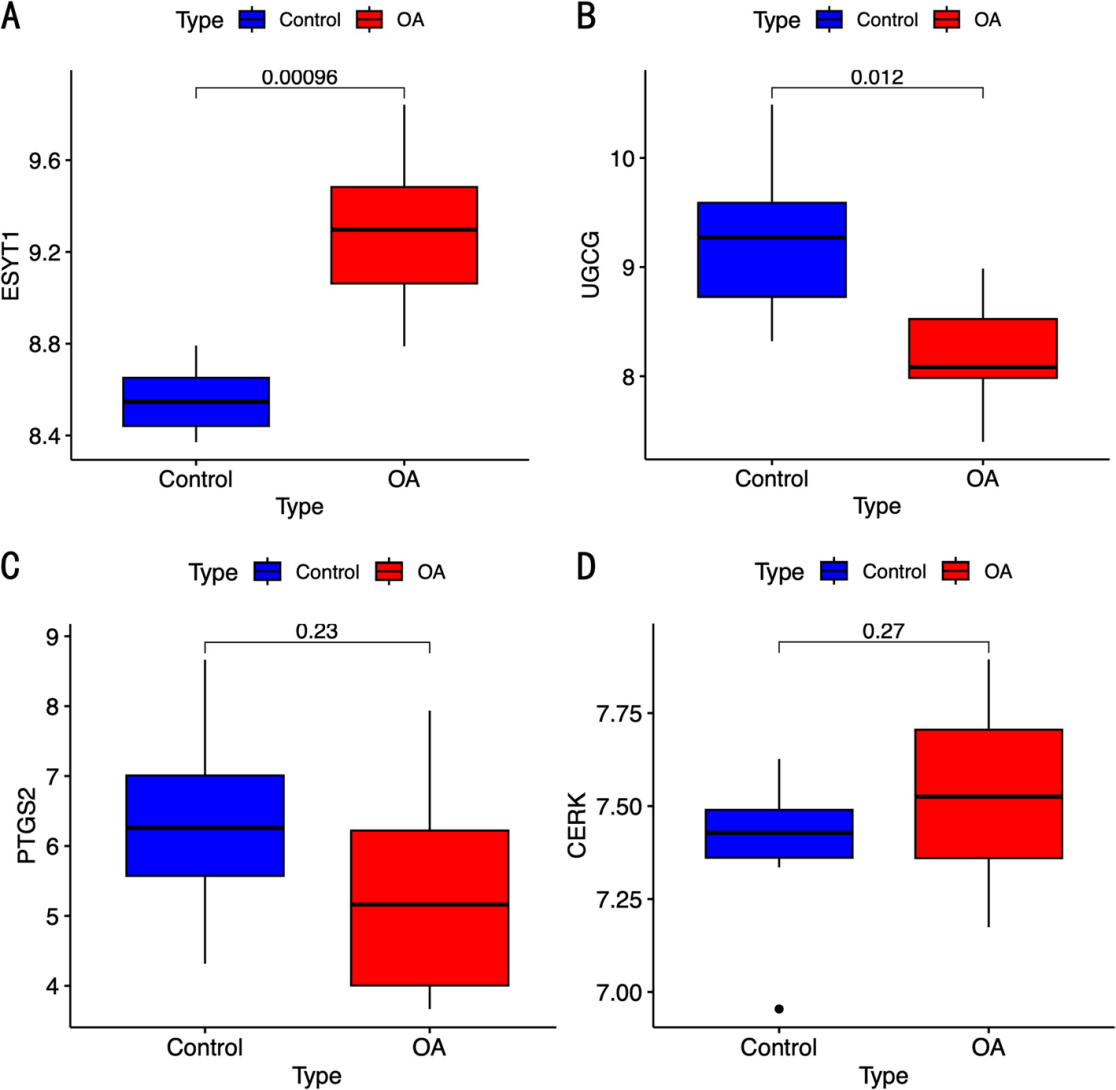
Validation set validation gene expression. **A-D **Box plot showing the expression of 4 hub genes in the validation dataset. *P* < 0.05 was considered statistically different when compared to the control group

The expression of the 4 osteoarthritis lipid metabolism hub genes in the validation dataset was visualized. Ultimately, ESYT1 and UGCG showed statistically significant differences in expression, and the disease group’s expression trend matched that of the GSE48556. ESYT1 expression was up-regulated in the disease group, whereas the expression of UGCG was down-regulated in the disease group (Fig. 6).

## Discussion

Osteoarthritis is a common chronic joint disease with pathologic changes occurring in the articular cartilage, subchondral bone, synovium, and joint ligaments. The prevalence of OA increases with age and more people will be affected by OA as human life expectancy rises. Genetic and environmental factors, including obesity, smoking, and nutrition elevate the risk of OA [14]. Research has indicated that 24.6% of knee pain cases can be attributed to overweight or obesity [15]. Mechanisms other than mechanical factors are unknown. In this study, we employed bioinformatics as well as machine learning algorithms to explore and validate osteoarthritis lipid metabolism genes in an attempt to provide new ideas for the pathogenesis of osteoarthritis from the perspective of lipid metabolism.

We obtained 363 osteoarthritis DEGs by extracting gene expression from the dataset GSE48556 of the GEO database and performing differential analysis. These DEGs were then analyzed by GO and KEGG enrichment, and the analysis results showed that they were mainly involved in biological processes such as myeloid cell differentiation, RNA splicing, and regulation of hemopoiesis. It has been suggested that gene transcription plays a role in the development of OA and may serve as its therapeutic target [16]. For instance, knockdown of the RNA-binding protein ZFP36L1 inhibited experimental OA in mice [17]. RNA variable splicing, RNA stability, transcription, and translation are the main types of genes that contribute to the development of OA [18]. RNA-binding proteins can regulate mRNA splicing in osteoarthritis, Hannah Swahn et al. found that the expression of RNA-binding proteins was higher than that of non-RNA-binding proteins in healthy cartilage, and that differentially expressed RNA-binding proteins were down-regulated to a greater extent in OA patients [19]. Hence, small molecule inhibitors targeting RNA-binding proteins may hold promise in OA treatment. In terms of cellular components, DEGs are mainly found in nuclear speckles, spliceosome complexes, and the nuclear membrane; in molecular functions, they are mainly enriched in ubiquitin-like protein ligase binding. Nuclear speckles serve as sites for the storage and modification of splicing factors, contributing to the integrated regulation of gene expression and bearing relevance to various human diseases [20]. It was demonstrated by Guifang Jia et al. that nuclear speckles in the nucleoplasm partially coexist with fat mass and obesity-associated protein (FTO) [21]. This also indicates a close relationship between RNA splicing and OA, which is consistent with the results of bioprocess analysis by GO analysis. KEGG enrichment analysis indicated that these genes were mainly activated in the chemokine signaling pathway, TNF signaling pathway, and C-type lectin receptor signaling pathway. The chemokine signaling pathway may be involved in osteoarthritis through inflammatory responses. These pro-inflammatory cytokines control adipocyte proliferation and death, encourage lipolysis, prevent lipid synthesis, and lower lipid levels via autocrine and paracrine processes. In addition, IL-1, TNF-α, and IL-6 can indirectly contribute to OA by regulating the release of lipocalin and leptin from adipocytes [22]. This shows that gene transcription and the inflammatory response may have a role in the development of OA.

After taking the intersection of 363 DEGs with lipid metabolism-related genes, we identified 9 hub genes. GO enrichment analysis of these 9 genes showed that they were related to lipid transport, organophosphate ester transport, lipid localization, glycerol, and phospholipid metabolism processes in biological processes. In terms of cellular components, these genes are mainly related to components of the peroxisomal membrane, nuclear membrane, and organelle membrane. At the molecular level, their functions were primarily related to lipid transporter activity, phospholipid transporter protein activity, and lipid transfer activity. It has been demonstrated that lipid deposition in chondrocytes leads to the development of OA and that dysregulation of lipid metabolism accelerates the development of OA [23]. The CH25H-CYP7B1-RORα axis of cholesterol metabolism in chondrocytes is an important mechanism involved in the development of OA, and the cholesterol content in OA chondrocytes is higher than that in normal chondrocytes [24]. Phospholipids contain long-chain fatty acids such as eicosapentaenoic acid and docosahexaenoic acid, which are essential components of cell membranes. In one study, patients with early and advanced OA had higher levels of most phospholipid species in their synovial fluid. Additionally, 66 phospholipid species had different levels in early OA than in late OA [25]. Phospholipids can also control the homeostasis of reactive oxygen species in the mitochondria [26] and have an anti- or pro-inflammatory effect [27]. Changes in phospholipid levels may therefore have an impact on the development of osteoarthritis (OA) by influencing joint lubrication, the removal of reactive oxygen species from joint fluids, and inflammatory responses in the joints.

Lipid mediators such as prostaglandins and leukotrienes are increased in the synovial fluid of patients with OA [28]. Additionally, plasma levels of interleukin-1, tumor necrosis factor-alpha, and interleukin-6, which are proinflammatory cytokines produced by immune-inflammatory cells in the adipose tissue, are significantly increased in OA patients [22]. F. Ponchel et al. found that in the peripheral blood of patients with OA, CD4 + T cells and B cells accounted for less, while the percentage of CD8 + T cells was higher than in normal subjects [29]. Moreover, there are inflammatory cells infiltrated in the synovium of OA, all of which suggest that there are immunoinflammatory factors in addition to mechanical stress factors in the development of OA [30]. Adipose tissue can secrete adipokines, which have an important role in the regulation of immune inflammation, and lipocalins can regulate obesity-induced inflammation in OA by modulating immune activity [31]. Therefore, obesity, inflammation, and immunity are inextricably linked to OA and create more possibilities for the diagnosis and treatment of OA [32].

After synthesizing three machine learning algorithms, Lasso regression, SVM-RFE, and random forest, four hub genes of osteoarthritis lipid metabolism were further screened: UGCG, ESYT1, PTGS2, and CERK. The expression levels of these four genes were verified by a validation set. In the end, it was found that two genes, UGCG and ESYT1, showed differential expression between the disease group and the control group, with the experimental set’s expression trends reflecting those of the disease group. The UGCG gene encodes UDP-glucose ceramide glucosyltransferase, representing the initial crucial step in the biosynthesis of the majority of glycosphingolipids. These lipids play a vital role as components within membrane microstructural domains, facilitating membrane translocation and signal transduction processes [33]. Naoki Seito et al. found that specific knockdown of UGCG in mouse chondrocytes resulted in more severe pathological OA induced by destabilization surgery and similarly more severe cartilage degradation induced by IL-1α in vitro experiments [34]. Loss of UGCG gene expression leads to increased ceramide levels in chondrocytes, subsequently leading to cartilage matrix degradation and apoptosis of chondrocytes [35]. Articular chondrocytes are the only kind of cells that constitute articular cartilage, and their functional changes are one of the important mechanisms for the development of OA [36]. Consequently, we hypothesized that the deletion of the UGCG gene may affect the development of OA by affecting the content of ceramide in chondrocytes. Therefore, more research is required to determine the precise mechanism.

ESYT1 belongs to the large family of ESYT proteins, which also includes E-Syt2 and E-Syt3 in mammals. It is more prevalent and pervasive in the brain [37]. Maintaining mitochondrial homeostasis and adaptation is a critical function of ESYT1, an endoplasmic reticulum-anchored protein with five C2 domains, an amino-terminal ER-membrane anchor, and a synaptotagmin-like mitochondrial lipid-binding protein domain (SMP) [38]. Lipids are mainly synthesized in the endoplasmic reticulum and then transported to mitochondria through the non-vesicular pathway of ER-mitochondria contact sites (EMCSs) [39]. Fei Kang et al. found that extracellular Ca2 + influx via the store-operated Ca2 + entry (SOCE) pathway mediated by endogenous stores of calcium ions activates ESYT1 to move toward the plasma membrane and that the activated E-syt1s themselves do not constitute membrane contact sites (MCS), but rather rearrange the adjacent ER structures into a circular MCS surrounding E-syt1, which helps to stabilize the MCS and accelerate local ER Ca2 + replenishment [40], suggesting that E-syt1 contributes to replenishing Ca2 + stores in the endoplasmic reticulum. The majority of E-syt1 research being done today focuses on tumors, and E-syt1 is a mediator of cell invasion in non-small cell lung cancer [41]. Kohji Yamada et al. showed that E-syt1 contributes to the growth of hepatocellular carcinoma cells and that it may also be a therapeutic target for hepatocellular carcinoma [42]. The onset of OA is closely associated with mitochondrial dysfunction [41], and overexpression of E-syt1 may result in lipid accumulation in the mitochondria, which aggravates mitochondrial dysfunction and contributes to the development of OA.

## Conclusion

In summary, our study integrated bioinformatics analysis and machine learning algorithms, revealing that lipid metabolism potentially plays a pivotal role in the pathogenesis and progression of osteoarthritis (OA). This involvement is through its influence on various processes, including transcriptional regulation, immune responses, and inflammatory responses, ultimately promoting cartilage matrix degradation and chondrocyte apoptosis. Moreover, we identified two hub genes, UGCG and ESYT1, which hold promise for introducing novel diagnostic perspectives in the context of OA.

### Supplementary Information

**Additional file 1.** The lipid metabolism gene list.

## Data Availability

Publicly available datasets were analyzed in this study. This data can be found here: (http://www.ncbi.nlm.nih.gov/geo/, https://www.gsea-msigdb.org/gsea/msigdb)

## Abbreviations

LMRGs: Lipid metabolism-related genes
DEGs: Differentially expressed genes
OA: Osteoarthritis
FC: fold change
GO: Gene Ontology
KEGG: Kyoto Encyclopedia of Genes and Genomes
SVM-RFE: Support vector machine-recursive feature elimination
LASSO: Least absolute shrinkage and selection operator
RF: Random forest
FTO: Fat mass and obesity-associated protein
SMP: Synaptotagmin-like mitochondrial lipid-binding protein domain
EMCSs: ER-mitochondria contact sites
SOCE: Store-operated Ca2 + entry
MCS: Membrane contact sites
